# Multi-facility virtual diagnostic for longitudinal phase space predictions

**DOI:** 10.1038/s41598-026-47195-1

**Published:** 2026-04-09

**Authors:** J. Lundquist, J. Björklund Svensson, P. Dijkstal, E. Mansten, G. Penco, S. Werin, F. Curbis

**Affiliations:** 1 Department of Physics, Lund University, Lund, Sweden; 2 PSI Center for Accelerator Science and Engineering, Paul Scherrer Institute, 5232 Villigen PSI, Switzerland; 3 MAX IV Laboratory, Lund, Sweden; 4 Sincrotrone Trieste, Trieste, Italy

**Keywords:** Engineering, Physics

## Abstract

A thorough understanding of the longitudinal phase space (LPS) of the electron beam is of great advantage to any modern linear accelerator (linac), and of critical importance for operating a free electron laser (FEL). While a transverse deflecting structure (TDS) allows full characterization of a beam’s LPS, measurements with a TDS system are often destructive and operationally complex. We present an application of machine learning in the form of a virtual diagnostic (VD) trained on destructive TDS measurements, which allows for online predictions of the beam’s LPS based on non-destructive measurements. We show the development and testing of such virtual diagnostics for three different accelerators: the MAX IV linac and the FELs FERMI and SwissFEL. We show how a single, general network architecture and training procedure can be used to reach reliable predictions of the LPS for all three facilities, achieving $R^2$ scores reaching 90% or higher across all test datasets. Further, we describe how a simplified architecture can be used for predicting key beam parameters of interest extracted from the full LPS, such as bunch length and slice energy chirp. Our results show how a generalizable VD framework can be rapidly deployed across multiple facilities to enable online monitoring of the beam LPS. For future work, we suggest how virtual diagnostics could be further developed to suit the specific needs of operations at each facility.

## Introduction

The use of machine learning in modern particle accelerators is a rapidly growing field. There is a wide range of applications possible with the varieties of machine learning methods currently being developed^1–6^. One such application with wide applicability is a virtual diagnostic (VD). Certain beam diagnostics are both crucial for setting up the accelerator and fully destructive to beam delivery, or can simply be time-consuming to calibrate and complex to use. One example of such diagnostics is the transverse deflecting structure (TDS), where radio-frequency (RF) fields are utilized to deflect the beam transversely in order to project the longitudinal profile of the beam onto one of the transverse planes^7^. TDS systems also include a spectrometer dipole to measure the energy distribution and a scintillating screen for producing an image of the full longitudinal phase space (LPS) of the beam. The goal of a full TDS system is often to provide information of the extent of the beam in the LPS, such as the bunch length and slice energy spread, whilst information of the beam’s first order moments, the beam centroid in energy and time, can be measured non-destructively using beam position monitors (BPM) in dispersive regions and beam arrival monitors (BAM).

The basic idea of a VD is to predict the measurements of destructive diagnostics like a TDS system using non-destructive information measured continuously in the rest of the machine. VDs have already been shown to provide reliable results at different facilities and in different contexts^8–11^. Based on such work, we have developed two types of VDs for this project: one type for predictions of full 2D LPS distributions and another for predicting critical beam parameters extracted from the full LPS. The second approach, focusing on critical beam parameters, provides a simpler network which can be trained in less time and using smaller datasets. In this paper we show the general applicability and relative simplicity in setting up and using these VDs for predictions of image-based destructive diagnostics. For many facilities, the use cases for the types of VDs developed in this project mostly overlap: to access online information about the beam’s LPS during delivery to experiments. With this purpose and use case, we have aimed to set up and test VDs at three different facilities that have not previously utilized image-based VDs, namely MAX IV, FERMI, and SwissFEL at PSI.

## Experimental setups

**Fig. 1:**
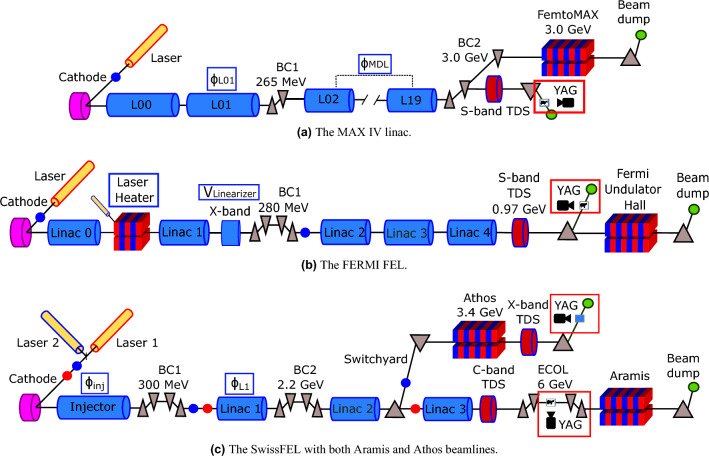
Simplified layouts of MAX IV (upper), FERMI (center), and SwissFEL (lower), indicating TDS locations along with YAG screen locations (red rectangles), as well as parameters scanned during data acquisitions (blue rectangles).

This study was carried out at three separate facilities (see Fig. 1), and the following section constitutes a brief description of each accelerator and its longitudinal diagnostic systems.

The MAX IV linear accelerator, shown in the schematic in Fig. 1a , can be largely divided into two accelerating sections. The first accelerating section raises the energy to 265 MeV. At this energy, the beam enters the first bunch compressor, BC1. MAX IV, unlike FERMI and SwissFEL, uses arc-like bunch compressors and does not use harmonic cavities to linearize the LPS before compression^12^. BC1 is followed by the main linac consisting of 32 separate accelerating structures accelerating the beam to the final energy of 3 GeV. The klystrons providing RF power to the main linac are fed from a common main drive line (MDL). After this, the beam enters a second bunch compressor, BC2, and can then be directed to two different beamlines, one being the experimental beamline FemtoMAX and one being the TDS line used in this study. The TDS being located in a separate beamline from FemtoMAX renders the TDS measurements incompatible with beamline delivery^13,14^. In the TDS beamline, the deflecting structures are followed by a dipole magnet, used to disperse the beam onto a YAG screen 20 m further down the beamline, where the beam is imaged. The YAG screen and relevant parameters for the MAX IV data acquisition are marked in Fig. 1a .

The FERMI linear accelerator, displayed in Fig. 1b , consists of four main accelerating sections, Linac 1-4^15,16^. The FERMI linac is also equipped with a laser heater system, which was set up to damp microbunching instabilities^17^. The bunch compression is performed using a magnetic chicane, BC1, in combination with an X-band linearizing RF structure. The RF phase and amplitude in the X-band structure have to be properly tuned to set the desired energy chirp in the electron bunch and linearize the bunch compression process. The diagnostic beamline at FERMI is in principle similar to the MAX IV system^18^. However, the S-band TDS is not located in a separate beamline from the normal delivery line, but is rather placed at the end of Linac 4. The beam can be deflected into a spectrometer line, where it is horizontally dispersed, allowing for reconstruction of the LPS. Thus, the measurement here is fully destructive to FEL delivery.

The SwissFEL is outlined in the schematic in Fig. 1c . The gun at SwissFEL produces two separate electron bunches within each RF pulse, using two laser pulses in the photo-cathode gun separated by 28 ns. This allows for parallel delivery to the two FEL beamlines, Athos and Aramis. After three stages of acceleration and two stages of bunch compression, the bunches are separated using a resonant kicker system at an energy of 3.1 GeV. The two bunches are then accelerated further in each beamline, to 3.4 GeV in Athos and to 6 GeV in Aramis^19,20^. The Aramis TDS is a C-band RF structure placed before the undulators^21^. The structure streaks vertically and is shortly followed by the energy collimator which contributes the dispersion for the LPS imaging. Since it is located before the undulators, this system is incompatible with continuous FEL delivery in the Aramis beamline. In the Athos beamline, the X-band TDS system^22,23^ is located after the undulators, thus in principle allowing for the diagnostic system to operate during X-ray delivery to the users of the Athos beamline, although only at a reduced beam repetition rate due to the increased radiation dose rate when the screen is inserted.

### Data collection and analysis

A critical step in any ML application is the collection of relevant and varied data. The choice of parameter space to investigate determines the extent to which predictions or classifications may be considered reliable and defines the use case for the application as a whole. With the goal of the VDs for all three facilities being to extract online information of the beam’s LPS, scans for data acquisitions were carried out with a similar structure between the facilities. Data acquired consisted of the destructive measurements from the TDS setup, along with all the relevant non-destructive information from the rest of the linac. Scans were performed around the normal operating point for the machine during delivery and the scanned parameters were the ones commonly used to adjust LPS shape as relevant for delivery. Further, scans were performed in a region sizable enough to cover a variety of different cases during operation. In all data acquisitions, 2D scans were used to densely and efficiently cover the parameter space. While random sampling may be more efficient in higher-dimensional parameter spaces^24^, the scanning method was considered sufficient for the parameters chosen. Further, the scanning method aligned with established data acquisition procedures at each facility and proved to be more practical, as the machine and diagnostics respond gradually to sudden changes in the machine state, which would occur if random sampling was used in exploring the parameter space. Below are brief descriptions of the performed scans for each facility, also summarized in Table 1.

**Table 1:** Parameters scanned at each facility, along with their corresponding ranges and number of steps.

Facility	Parameter	Range	Steps
MAX IV			
	L01 phase	$\pm\,\, 3.97^\circ$	30
	MDL phase	$\pm\,\, 3.18^\circ$	30
	L01 filltime	$\pm\,\, 0.1\,\upmu \text {s}$	–
	MDL filltime	$\pm \,\,0.25\,\upmu \text {s}$	–
FERMI			
	LH laser energy	0–$10\,\,\upmu \text {J}$	20
	BLM reading	$1.5\cdot 10^{6}$–$4\cdot 10^{6}$	20
SwissFEL			
	Injector phase	$\pm \,\,0.2^\circ$	$\approx 18$
	Linac 1 phase	$\pm \,\,0.3^\circ$	$\approx 18$

During the MAX IV data acquisition, scans were performed on two parameters commonly used for bunch length trimming: the RF phases in the pre-BC1 accelerator L01 and in the structures along the MDL. To maintain full transmission through the bunch compressors these phase scans were in practice carried out by scanning the SLED filltime, i.e., the time spent by the RF pulse in the SLED RF pulse compressors^25^. This will be directly connected with the amplitude of the RF field experienced by the passing beam. RF phases were then scanned by letting feedback alter the relevant phases to maintain the final energy. The ranges and numbers of steps of both these correlated parameters are shown in Table 1. For each step in the 2D scan, 10 measurements were taken, resulting in a final dataset of 9000 images and corresponding non-destructive information, consisting of RF phases and SLED filltimes, as well as readings from BPMs and current transformers.

During the FERMI data acquisition, the linac was set up with only one bunch compressor and the scanning procedure was thus somewhat modified from the MAX IV case. The one bunch compressor that was in use is also of the more common chicane type combined with a linearizing X-band cavity, as compared with the arc-like compressors of the MAX IV linac^12^. In order to keep a constant bunch compression factor, a feedback reads a bunch-length monitor (BLM) and acts on the RF phase of the X-band linearizer. For this measurement we scanned the target BLM reading of this feedback to change the final bunch length. As a second scan parameter, the pulse energy of the laser heater was varied, allowing for changes to the slice energy spread^17^. The ranges of these parameters are shown in Table 1. For each step in the scan 10 measurements were taken, resulting in a final dataset used for training and testing networks of 4000 images, along with RF phases and amplitudes, and measurements from BPMs, a BAM, the BLM and the laser heater pulse energy.

In the SwissFEL linac the use of two bunch compressors allowed for a parameter scan similar to the one carried out at MAX IV. The scanned parameters were the phases of the injector stage of the linac before the first bunch compressor and the phase of Linac 1. As these scan parameters affect both bunches, i.e., the bunches going to Aramis and Athos, a simultaneous data acquisition was performed from the TDS systems in both beamlines. The data acquisition time was limited by the Aramis line, as the TDS setup is located in a region of the tunnel with strict radiation dose limits, and smaller scans were performed at separate times to comply with integrated dose limits. Because of these separate scans, the stepsize was not always homogeneous, but the final dataset consisted of approximately an $18\times 18$-steps scan of the total region. The scanned parameters, their ranges and approximate number of steps, as shown in Table 1, are also the same for both beamlines. For each step, 5 measurements were collected from both TDS systems, resulting in a total of approximately 1600 images from each TDS system, along with RF phases and amplitudes, and measurements from BPMs, a BAM and two bunch compression monitors.

For each facility, the listed data collections were performed in a single day. For a more comprehensive stability analysis of the implemented VD methods, a separate data acquisition campaign was carried out at MAX IV. This consisted of recurring data collections over five days to allow the machine state to drift and observe the effects on VD predictions. An initial 2D scan of SLED filltimes was performed to set up and train the VD, and on the subsequent four days, approximately 100 images were collected per day at the nominal machine setpoint as separate datasets to test the VD on, without additional training. The initial training dataset consisted of 400 setpoints with three images per point, covering a similar region to the larger dataset explained earlier, with filltime ranges of $\pm 0.15$ $\upmu {\rm s}$ and $\pm 0.125$ $\upmu {\rm s}$ in the L01 and MDL SLED pulse compressors respectively.

## Results

Below we present the results for two types of networks, i.e. full LPS and beam parameter predictions, for all four accelerator beamlines. In all cases, the results presented are those on the test datasets, separated from the original datasets before the training procedure.

### Full image predictions

**Fig. 2:**
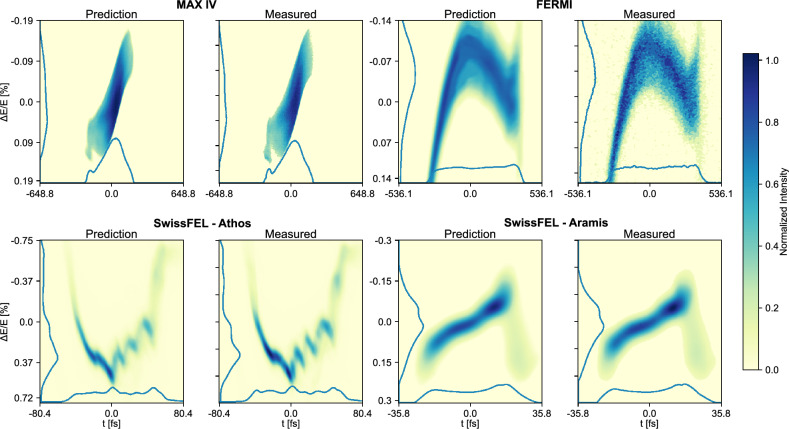
VD predictions and measured results of the LPS measurements at the **median** performance in terms of $R^2$, as highlighted in Fig. 7, for the four cases.

Examples of the full LPS predictions are displayed together in Fig. 2. These examples were chosen at the median performance for each dataset in terms of $R^2$ (see“Scoring Method” section in Methods, Eq. 1). Overall, the similarity between the VD predictions and the measured images is good. Beyond this, one may also note that the VDs accurately predict some of the finer structures, such as in the Athos case, and in the tails of the FERMI and Aramis cases. Further, there is a clear similarity between predictions and measurements of the profiles (energy and time), projected down onto each of the axes in all images.

**Fig. 3:**
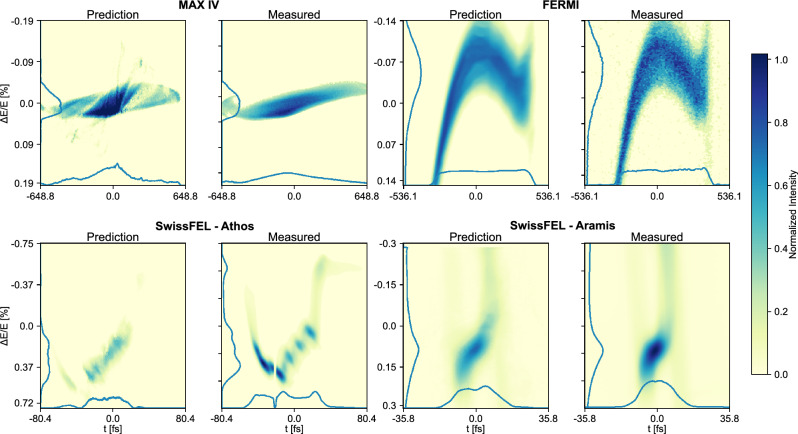
VD predictions and measured results of the LPS measurements at the **worst** performing cases in terms of $R^2$.

The median performance is high in all four datasets, but on the lower end of $R^2$ there are outliers and these are shown in Fig. 3. The worst outliers occur in the MAX IV and Athos datasets. In the MAX IV case there are no clear artifacts or other issues with the measured image, and the poor prediction may be due to an issue with the input from the non-destructive diagnostics to the VD. For the purpose of this study, all data has been included, but for future, online VD applications it may be of great interest to label or remove data points with divergent input values, as compared with the training dataset. For Athos, there is a minor artifact in the core of the beam which could have affected the training for beams measured on this specific area of the screen, although there could also have been an issue in the non-destructive information for this measurement.

**Fig. 4:**
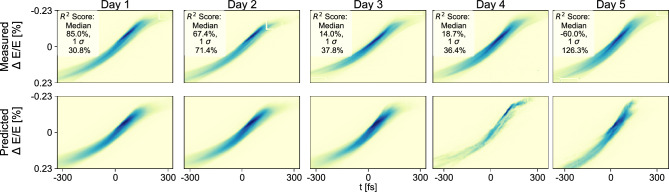
Median predictions and corresponding measured images in terms of $R^2$ scores for a model trained on Day 1 and tested on data over the subsequent four days.

The results from the MAX IV stability study are shown in Fig. 4, with median image predictions and their corresponding TDS measurements, chosen at the median $R^2$ score from each dataset. The median $R^2$ score is also printed and can be seen to rapidly decline from Day 2 onwards, but maintains some stability until a sharper decline in the Day 5 dataset. One can also observe a breakdown in predictions in the median images from Day 4 onwards. While the measured LPS remains similar over all days, the machine state may have shifted enough by Day 4 to cause this breakdown. Some parameters, such as the on-crest RF phases and the calibration of the TDS system, will drift and remain unseen by the network. All the same, the VD could maintain acceptable LPS predictions over a few days without any extensive work in extending this stability. One can also note that this data was collected during time allocated for studies of the MAX IV linac, during which the machine state is adjusted more frequently and drastically as compared with nominal delivery.

### Beam parameter predictions

When working with LPS measurements for delivery to users, the main objective is often to achieve precise tolerances in a set of key parameters that are relevant for that delivery. Rather than predicting full LPS images, it may be sufficient to predict the specific parameters of the LPS distribution. For this purpose, we set up separate networks and trained them to predict only the most relevant aspects of the collected LPS images. These parameters were chosen as the slice energy spread in the beam center, the full energy spread and the bunch length of the beam, and the energy chirp. For the energy spreads, RMS values on the relevant distributions were used. For bunch lengths, RMS was also used for the MAX IV distributions, but for FERMI and SwissFEL FWHM values were chosen due to the non-Gaussian shape of the bunch profiles. Energy chirp calculations were performed using polynomial fits to the center of the chosen segments of the beam. The order of chirp to predict was chosen based on the typical shapes of the chirp for each application: first order for MAX IV and second order for the FERMI and SwissFEL cases.

**Fig. 5:**
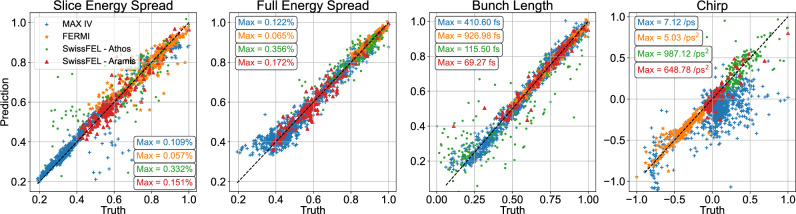
Correlation plots of the beam parameter predictions in each dataset. The beam parameters, both measured and predicted, were normalized by the maximum of the measured values displayed in color-coded text in each plot. The different units in the chirp RMS results from the use of first or second order chirp.

**Fig. 6:**
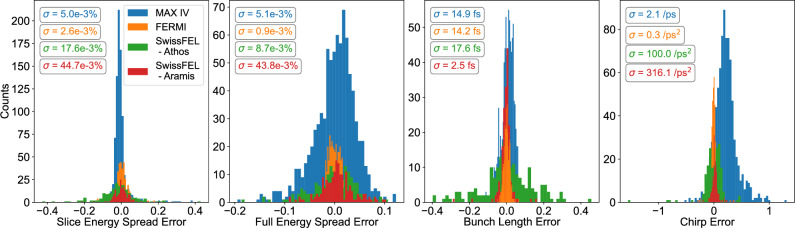
The error distributions in the beam parameter predictions binned into histograms with the RMS of each distribution displayed. The error distributions were calculated from the normalized values shown in Fig. 5, while the RMS is displayed in the non-normalized units. The different units in the chirp RMS results from the use of first or second order chirp.

The normalized results of the beam parameter predictions are shown in Fig. 5, here in the form of correlation plots, with the predicted values along the vertical axes and the ground truth along the horizontal. The maximum values in the data used for normalization are also shown for each parameter and dataset. These plots are presented along with the 1:1 line to show the ideal predictive case; any deviations from this line are errors in the predictions by the VD. These errors were calculated and binned into histograms, displayed in Fig. 6 along with the RMS, $\sigma$, of each distribution. Here again, the errors are based on normalized calculations while the RMS is displayed in the true units. There are mostly strong correlations between prediction and ground truth, e.g., the RMS errors in the bunch length predictions are on the order of 10 fs or below for all four cases, which would be a high resolution to reach with most actual TDS measurements^26^. We see some larger errors in the predictions of energy chirp in the case of MAX IV. This is most likely connected with the higher variance in this dataset as compared with the other three, as mentioned above. The chirp, extracted in this case as the linear component, changes sign during the parameter scan, which complicates the predictions.

For a comparison between the networks used for full LPS prediction and the VDs focused on beam parameters, one can look at the final achieved Pearson correlation coefficient, *r*, between predicted and measured beam parameters in both cases, with the beam parameters for the full LPS VD being extracted from the final predicted LPS distributions. These coefficients, for each facility, network type and parameter, are summarized in Table 2. We can see how in most cases the performance of the VDs is quite comparable. Outliers exist, especially in energy chirp predictions. The beam parameter VD performs quite poorly in the case of the beam chirp in Athos, whilst the full LPS performs to a more satisfactory degree. This may be due to the complex shape of the Athos beam’s LPS, while we see the beam parameter VD performing better in the Aramis case, where the beam distribution is comparatively simpler. Energy chirp is inherently dependent on the full shape of the beam’s LPS, and it appears to be advantageous to have the full image available to the network if this parameter is of interest.

**Table 2:** Pearson correlation coefficients for each beam parameter and facility, correlating between measured and predicted values, either using the beam parameter VD and the full LPS VD to predict the beam parameters.

Facility/VD	*r*$_{{\rm Slice}, \sigma _{\rm E}}$ [%]	*r*$_{{\rm Full}, \sigma _{\rm E}}$ [%]	*r*$_{\sigma _{\rm z}}$ [%]	*r*$_{{\rm Chirp}}$ [%]
MAX IV				
Beam Param.	96.4	97.0	98.9	72.4
Full LPS	99.0	99.5	95.3	82.3
FERMI				
Beam Param.	93.1	94.8	99.3	92.9
Full LPS	82.0	99.6	97.7	92.1
SwissFEL - Athos				
Beam Param.	79.2	90.0	85.7	48.7
Full LPS	78.5	79.6	83.4	92.0
SwissFEL - Aramis				
Beam Param.	94.1	96.6	97.0	90.7
Full LPS	95.3	93.0	98.5	77.5

## Discussion

The implementation of a general machine learning application with a standardized training procedure for image-based predictions at multiple facilities was developed and tested, along with a simpler variant for beam parameter predictions. Data acquisitions were carried out at three different facilities, MAX IV, FERMI and SwissFEL, with a parallel acquisition in two streaking systems at once carried out in the SwissFEL case. Scans of parameters connected with LPS shaping for normal delivery were carried out. For each facility and dataset, networks were trained to predict both full LPS images and and beam parameters extracted from the LPS images. The networks set up for predicting beam parameters had a simpler architecture and were significantly faster to train. The predictions show generally good performance for all four datasets, for both the image and beam parameter cases, with a relatively small number of outliers. This demonstrates the potential use of VD systems in these and other facilities for the online monitoring of LPS distributions, where such measurements would otherwise be destructive or time-consuming. At MAX IV, a separate experiment was carried out over a full week of studies to test the stability and reliability of pre-trained VD applications. While this showed a clear deterioration of performance over a week of drifts in the machine state, the VD could still maintain reliable predictions over multiple days. With further work to promote VD stability, such as expanding the inputs available, this period of stability could most likely be extended. Since data acquisition and network training can be completed in a single day using standardized procedures, a new VD could then be deployed as needed to compensate for the long-term drifts or accelerator changes that render an existing, pre-trained VD unstable.

Naturally, another non-destructive way to probe the LPS of the beam is to perform highly-detailed conventional simulations of the machine. In comparison with simulation methods, such as particle tracking or particle-in-cell codes, a pre-trained VD will offer significantly reduced computational cost and time. The improvement in computation time, from minutes or hours for simulation to milliseconds for VDs, enables online applications of the results, such as use in optimization or feedback loops. With sufficient training data, the VD may also predict dynamics and structures which are difficult to model accurately; since the predictions are based on real data it will implicitly capture collective effects such as wakefields and coherent synchrotron radiation.

The work carried out for this article has focused on the testing of VD systems in facilities where previously this approach was not used. For future work, the predictions by VD systems such as these should be tested on separate datasets and on data collected online during operations. Future data acquisitions may also investigate the use of active learning ML systems to build on the acquisitions using scans in this study^27^. This would involve using ML to select critical setpoints in the machine for a more varied and robust dataset. VD systems of similar scope and structure could be set up through the same process at any number of facilities hereafter. An interesting area to explore further could also be transfer learning between facilities, whether general accelerator dynamics can be learned in a network to then be fine-tuned with facility dependent training runs. Beyond this, there are separate use cases for each facility which should be investigated further: at MAX IV there is an interest in expanding the TDS resolution by combining training datasets with higher resolution simulations, at FERMI a VD that could span multiple beam energies is under consideration, and for SwissFEL there is the potential for applications involving photon diagnostics and predicting FEL pulse power profiles.

## Methods

### TDS calibration

For the proper longitudinal coordinates to be determined, a calibration of the streaking performed by the deflecting structures is required. The calibration of a TDS is commonly done through a phase scan of the streaking RF field^28,29^. As one alters the phase angle of the deflecting field, one gets different angles of deflection and resulting centroid locations on the end-of-line screen. This operation provides a calibration factor of the transverse coordinate in terms of position on the screen relative to the deflecting RF phase angle, ${\frac{{\rm m}}{\text {deg}}}$, and with the frequency of the RF field one can transform it to ${\frac{{\rm m}}{\text {s}}}$. These scans were performed before each measurement carried out for this work, and the resulting calibration factors are summarized below.

For the MAX IV linac, a phase scan of the deflecting structures was carried out, resulting in a calibration factor of 2.92 $\frac{{\rm mm}}{\text {ps}}$, and for the energy calibration, a calculated dispersion value of 1.016 m was used. The calibration for the FERMI TDS, performed with a similar method as above, for this study gave a factor 2.33 $\frac{{\rm mm}}{\text {ps}}$ and the local dispersion at the screen was calculated to 1.716 m. For the SwissFEL Aramis TDS, the calibration factor attained at the time of the data acquisition was 30.82 $\frac{{\rm mm}}{\text {ps}}$ and the dispersion at the screen was calculated as 0.878 m. At the time of the measurement, a calibration factor of 41.2 $\frac{{\rm mm}}{\text {ps}}$ was attained for the Athos TDS and the dispersion at the screen was calculated as 0.177 m.

### Image and input preparation

A critical step for the machine learning applications described in this paper is the preparation of the images and input vectors for use in the artificial neural networks (ANNs). The processing procedure for the images has similar steps for each of the separate applications. The first two steps consist of a background subtraction and applying a median filter over the image. Following this, it was necessary to crop the image, both to isolate and center the beam on the images later used in the networks in order to improve both VD performance and training times. Cropping was done using *scikit* region detection, which isolates separate structures within the image using some given threshold^30^. In the case of the applications covered here, Otsu’s method was used to find a distinct threshold between background and relevant structures^31^. The beam was then chosen among the image structures as the structure with the largest product of area and intensity. Cropping of the images was then done to a region-of-interest centered on the beam.

The non-destructive information from the data acquisitions consisted mainly of RF settings and signals from BPMs in dispersive sections of each machine. In the case of MAX IV, current transformers were also included, and for both FERMI and SwissFEL beam compression monitors were available. In order to use this data for training the VDs, normalization is required. The input vectors were in all cases a collection of different non-destructive readings and setpoints throughout the machines with widely different ranges and variances. As such, different groups of inputs were normalized separately. Each group was normalized by a physically informed constant value throughout the datasets, e.g., transverse BPM signals were normalized by the reading of 1 mm and RF phases by 360$^\circ$.

### Networks and training

The ANNs developed for this project utilized mainly two common types of ANN structures. These were dense layers and convolutional neural networks (CNNs)^32^. CNNs are networks specifically constructed to work on matrix inputs. The trainable weights are applied to the data as a small filter moving across an input matrix, rather than the weighted connections used in the dense layers. For the ANNs presented in this work, the loss function was either mean absolute error or mean squared error, and the optimizer was the ADAM algorithm, commonly utilized for ANN training^33^. All ANNs were set up using the TensorFlow library for Python^34^. Before any training, the data was always set in a random order and 10% of the dataset was separated before training for testing the network’s generalized performance after training. Another 10% was separated during training into a validation set used to test generalized performance during the training iterations.

#### Full longitudinal phase space predictions

The networks utilized for predicting the full LPS images were of a vector-in matrix-out type, working with a combination of dense layers and CNNs. First came two dense layers, each of 100 nodes, and between these layers a step of batch normalization was implemented^35^. A final dense layer was required with the same number of nodes as the size of the final images. This layer, while critical to reach the high-resolution image results, was thus quite large, e.g., 422,500 nodes in the MAX IV case, and extended training times significantly. After this, the large output from the final dense layer was reshaped into matrices and put through two CNN layers, the first consisting of four 4$\times$4 kernels and the second of one 5$\times$5 kernel. This specific structure was a result of iterative improvements. Simpler networks which did not include the CNN layers were tested but showed worse performance. Conversely, more complex networks with further layers added were tested as well as other network types, but found to only marginally increase performance.

Throughout this network, the Rectified Linear Unit (ReLU) activation function was used, defined as $\textit{y}(\textit{x})=~(\textit{x}+|\textit{x}|)/2$. The networks were trained with the functions mentioned in the previous section, with a learning rate factor that was updated throughout training to fine-tune the performance. Network weights were saved throughout training and after the final iterations the weights that minimized the loss in the validation dataset were applied as the final network. As a final step, predicted distributions of the LPS were centered on the ground truth distribution. This was done by cross-correlating the two images and then performing an affine transformation on the predicted image, shifting it to the point of maximum correlation with the ground truth. The number of trainable parameters varied between datasets, due to the variable size of the images, but remained on the magnitude of 10 million parameters. Training when iterating on the networks was done on an NVIDIA A100 GPU, with training times reaching approximately 10 minutes. Training times on an 8-core laptop CPU were on the scale of 1–2 h.

#### Beam parameter predictions

The networks used for predicting the beam parameters were naturally simpler than the networks used for full LPS predictions. These consisted only of dense layers, the first two of 250 nodes each, then a final layer of four nodes for output. Here, batch normalization was also utilized between the 250-node layers^35^. The activation functions of the first two layers were hyperbolic tangent functions, and a simple linear activation was used in the final layer to allow for negative outputs. The same steps of scheduled learning rate and loading of ideal weights were applied during these trainings. The final networks used for beam parameter predictions had a total of approximately 75,000 trainable parameters. Training times for this type of network stayed at approximately 3 minutes on an 8-core laptop CPU.

### Scoring method

**Fig. 7:**
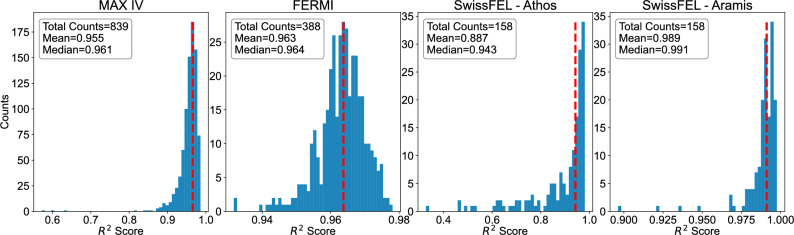
$R^2$ score for test datasets for each facility binned into histograms, also displaying the total counts, mean and median of each distribution. The dashed red line marks the sample performance used for plotting in Fig. 2.

To have a numerical measure of performance on full LPS predictions, we applied to each dataset with image predictions the scoring method of coefficient of determination, or $R^2$, defined as follows:

1 $$\begin{aligned} R^2=1- \frac{\sum _\textit{i}(\textit{y}_\textit{i}-\textit{x}_\textit{i})^2}{\sum _\textit{i}(\textit{y}_\textit{i}-\bar{\textit{y}})^2}, \end{aligned}$$

where $\textit{y}_\textit{i}$ is the ground truth, $\textit{x}_\textit{i}$ is the predicted value and $\bar{\textit{y}}$ is the mean of the ground truth. The performances in terms of $R^2$ across the full test datasets are displayed in Fig. 7 binned into histograms. Here we can see the generally high performance across all datasets, reaching both median and mean performances of above 95% for the MAX IV, FERMI and Aramis cases. For FERMI and the Aramis line of SwissFEL we see nearly all setpoints in the test dataset reaching above $R^2=90\%$. There are a few outliers in the cases for MAX IV and the Athos SwissFEL beamline. The MAX IV dataset is larger and more varied than the other datasets, and as such, some more extreme cases may be harder for the VD to predict. Similarly, the variation in the Athos set was higher than in the Aramis set, since the scan was set up close to the point of maximum compression.

In the MAX IV case, there are no clear artifacts or other issues with the measured image, and the poor prediction may be due to an issue with the non-destructive input to the VD. For Athos there is a minor artifact in the core of the beam which could have affected the training for beams measured on this specific area of the screen, although there could also have been an issue in the non-destructive information for this measurement.

## Data Availability

The datasets generated and analyzed during the work are available from the corresponding author on reasonable request.
